# Supplementary material for: A rationally designed miniature of soluble methane monooxygenase enables rapid and high-yield methanol production in Escherichia coli
Source: Nat Commun. 2024 May 23;15:4399. doi: 10.1038/s41467-024-48671-w (PMC11116448; doi:10.1038/s41467-024-48671-w)
Supplement: Supplementary file 1 — Supplementary Information [file 41467_2024_48671_MOESM1_ESM.pdf]

Supplementary Information for

A rationally designed miniature of soluble methane monooxygenase enables rapid and high-yield methanol production in *Escherichia coli*

Yeonhwa Yu[1], Yongfan Shi[2], Young Wan Kwon[3], Yoobin Choi[1], Yusik Kim[1], Jeong-Geol Na[2], June Huh[1]* & Jeewon Lee[1]*

[1] Department of Chemical and Biological Engineering, Korea University, Anam-Dong 5-1, Seongbuk-Gu, Seoul 02841, Republic of Korea.

[2] Department of Chemical and Biomolecular Engineering, Sogang University, Seoul, 04107, Republic of Korea.

[3] KU-KIST Graduate School of Converging Science and Technology, Korea University, Anam-Dong 5-1, Seongbuk-Gu, Seoul 02841, Republic of Korea.

* Corresponding authors: junehuh@korea.ac.kr; leejw@korea.ac.kr

Supplementary Information Inventory:

Supplementary Figures 1-18

Supplementary References

Supplementary Figures

a

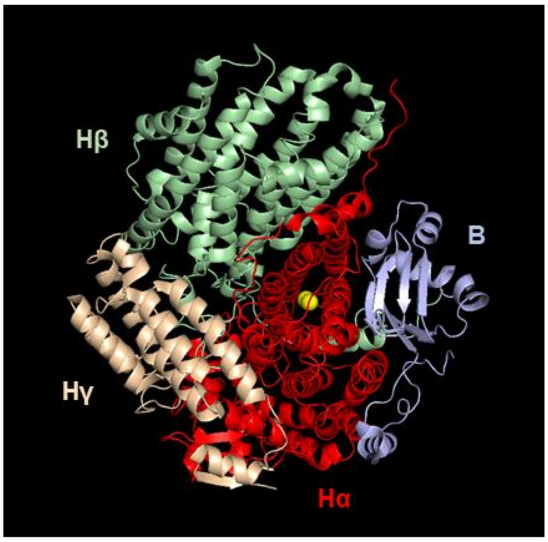

b

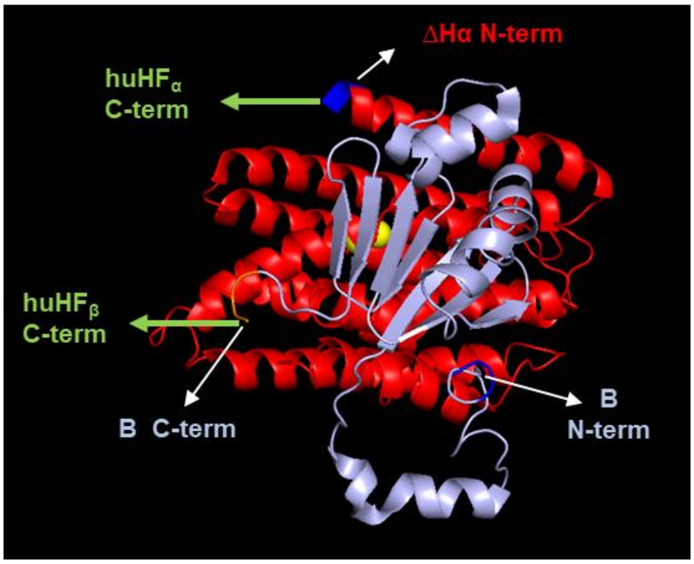

Supplementary Figure 1 | Native MMOH-MMOB complex structure. (**a**) The crystallographic complex structure of native MMOH (αβγ) protomer (Hα (red), Hβ (green), Hγ(wheat)) and MMOB (light blue) (PDB ID code: 4GAM). Yellow colored spheres represent diiron. (**b**) The crystallographic complex structure showing only the sub-structure of native MMOH (corresponding to ΔHα (red)) and MMOB (light blue) in (a), showing the both the N-termini (blue) of ΔHα and MMOB are facing the opposite direction. The green arrows indicate that the C-terminus (orange) of MMOB is linked to the C-terminus of huHF subunit (huHF$_\alpha$), while the N-terminus of ΔHα is linked to the C-terminal direction of another huHF subunit (huHF$_\beta$) to maintain the native direction of MMOH and MMOB in the complex structure.

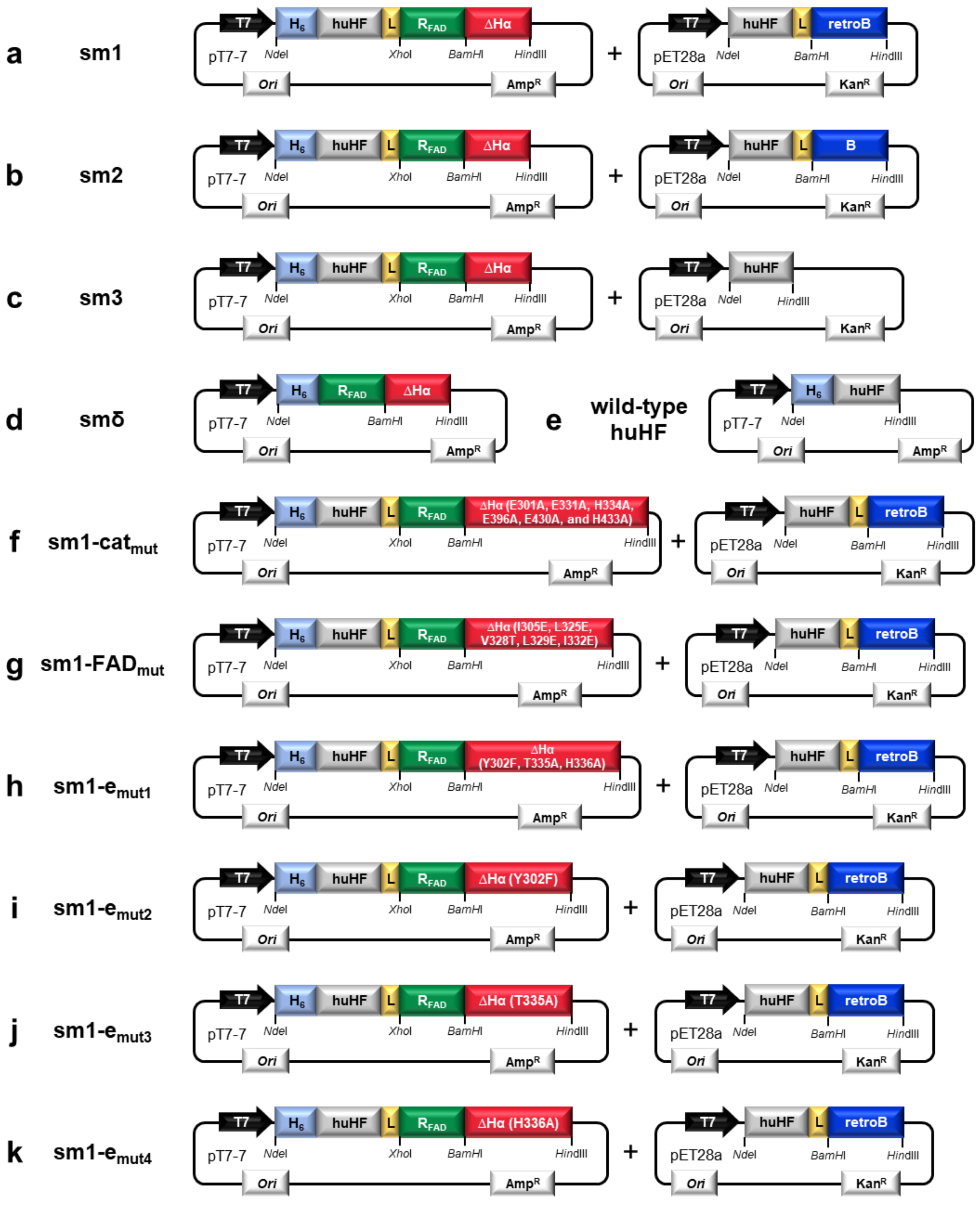

Supplementary Figure 2 | Plasmid expression vectors used for synthesizing sm1 to sm3, sm1-derived mutants, smδ, and wile-type huHF in recombinant *E. coli*. pT7-7 and pET28a plasmid backbone-based expression vectors including T7 promoter, replication origin (*ori*), antibiotic-resistance gene (Amp^R/Kan^R), and the coding sequence for synthesizing of (**a**) sm1, (**b**) sm2, (**c**) sm3, (**d**) smδ, (**e**) wild-type huHF, (**f**) sm1-cat$_{mut}$, (**g**) sm1-FAD$_{mut}$, (**h**) sm1-e$_{mut1}$, (**i**) sm1-e$_{mut2}$, (**j**) sm1-e$_{mut3}$ and (**k**) sm1-e$_{mut4}$. Except for the synthesis of smδ and wile-type huHF, *E. coli* was transformed with the two plasmid expression vectors to synthesize sm1 to sm3 and various sm1-derived mutants.

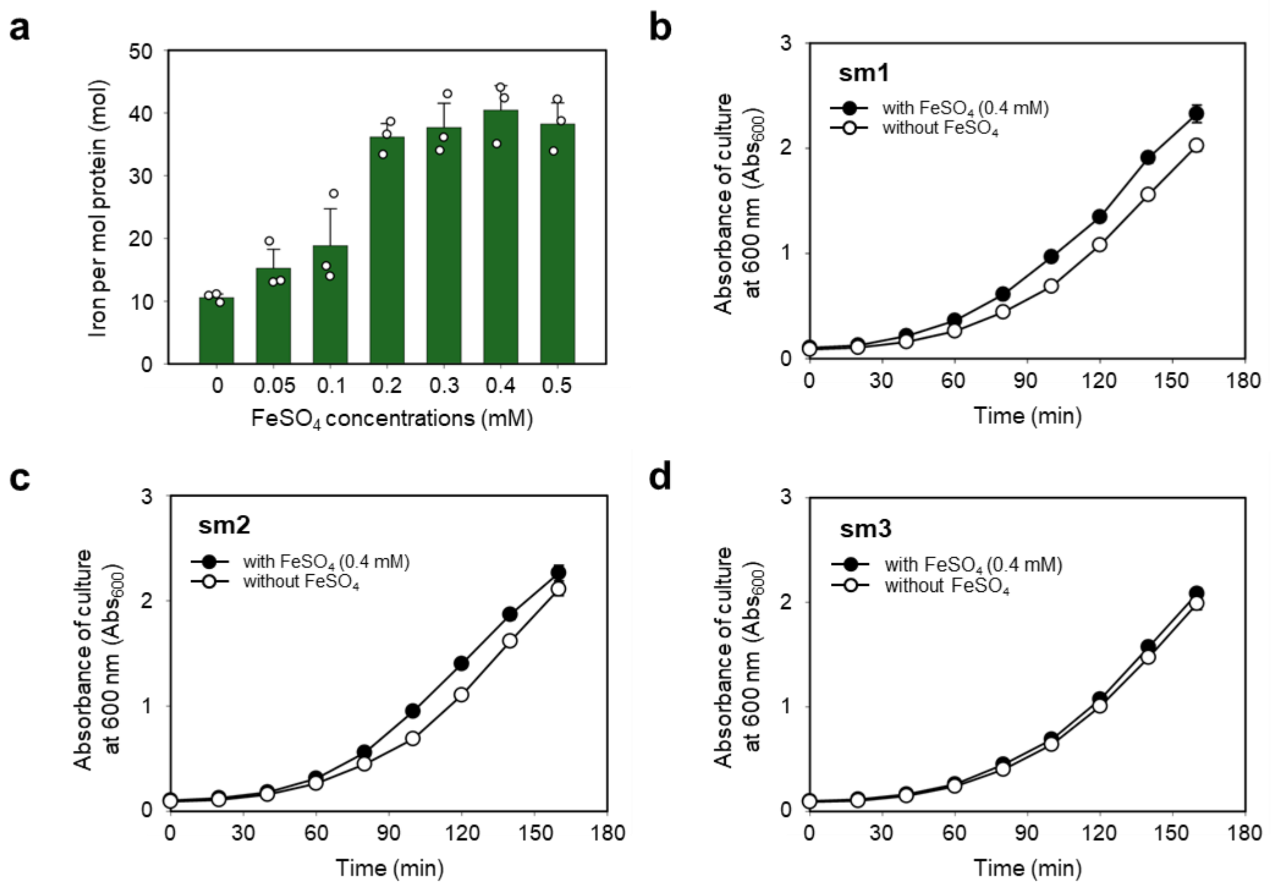

Supplementary Figure 3 | Effect of $FeSO_4$ (0.4 mM) in the medium on the growth of mini-sMMO (sm1 to sm3)-expressing recombinant *E. coli*. (a) Variation in iron quantity (moles) loaded on a mole of sm1 synthesized in *E. coli* as the concentration of iron sulfate in the culture medium increases up to 0.5 mM. $N = 3$ independent experiments. Mean ± s.d. **(b-d)** Time-course growth of the recombinant *E. coli* producing mini-sMMOs (sm1 to sm3), when the growth medium is supplemented with $FeSO_4$ (0.4 mM). All measurements were done three times at a time (Mean ± s.d.)

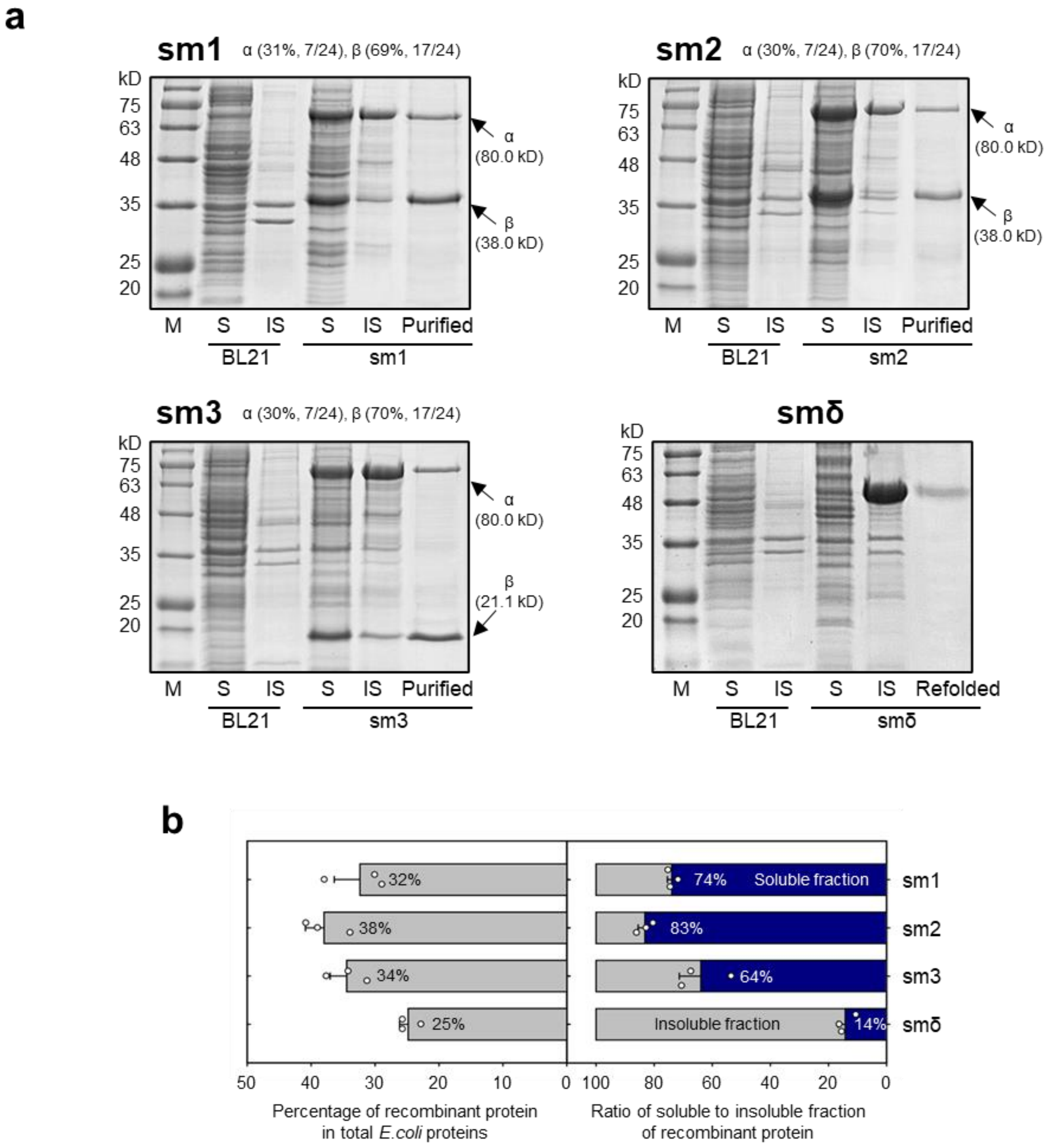

Supplementary Figure 4 | Heterologous expression of mini-sMMOs and smδ in *E. coli*. (**a**) Results of SDS-PAGE showing the expression level and cytoplasmic solubility of expressed mini-sMMOs (sm1 to sm3) and smδ. (M: protein marker (GangNam-STAIN), S and IS: soluble and insoluble fraction of expressed protein, respectively, Purified: mini-sMMO eluted from the Ni^{2+}-affinity purification column loaded with soluble fraction, Refolded: smδ purified through the refolding step of insoluble aggregates, BL21: wild-type *E.coli* BL21(DE3)). This experiment has been repeated at least three times (**b**) The expression level (left) and the cytoplasmic solubility (right) of mini-sMMOs (sm1 to sm3) and smδ. $N = 3$ independent experiments. Mean ± s.d.

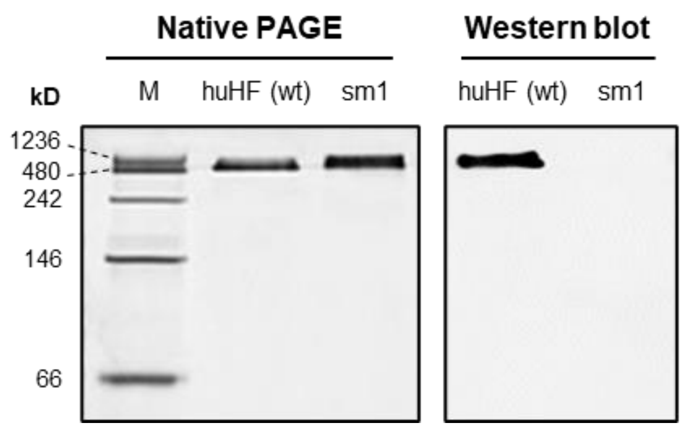

Supplementary Figure 5 | Native PAGE and Western blot analysis of sm1 and wild-type huHF. Results of Native-PAGE (left) and Western blot (right) of the purified wild-type huHF and sm1. For Western blotting, anti-huHF IgG was used as a primary antibody (Methods). (M: native protein marker). This experiment has been repeated two times.

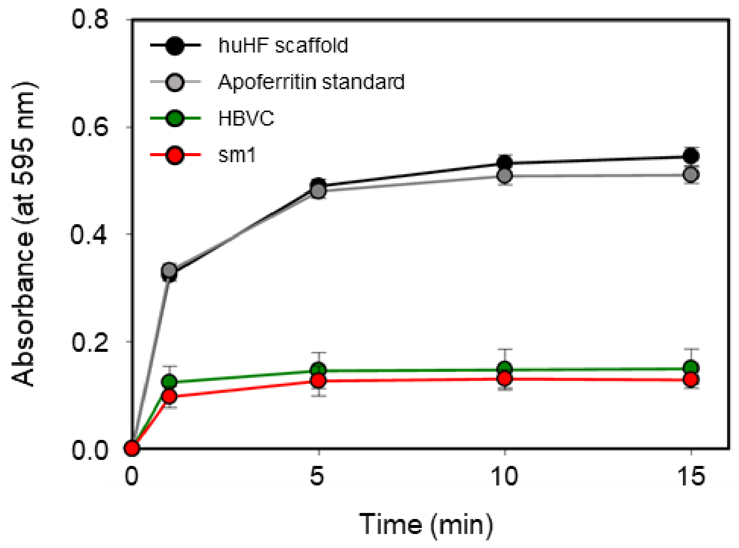

Supplementary Figure 6 | Ferroxidase activity estimated through xylenol orange (XO) assay. Ferroxidase activity was assayed by adding 40 μM $(NH_4)_2Fe(SO_4)_2$ to each sample (huHF scaffold, apoferritin standard, HBVC, or sm1) at 0.8 μM, followed by measuring the absorbance of Fe^{3+}-XO complex at 595 nm. $N = 3$ independent experiments. Mean ± s.d.

a

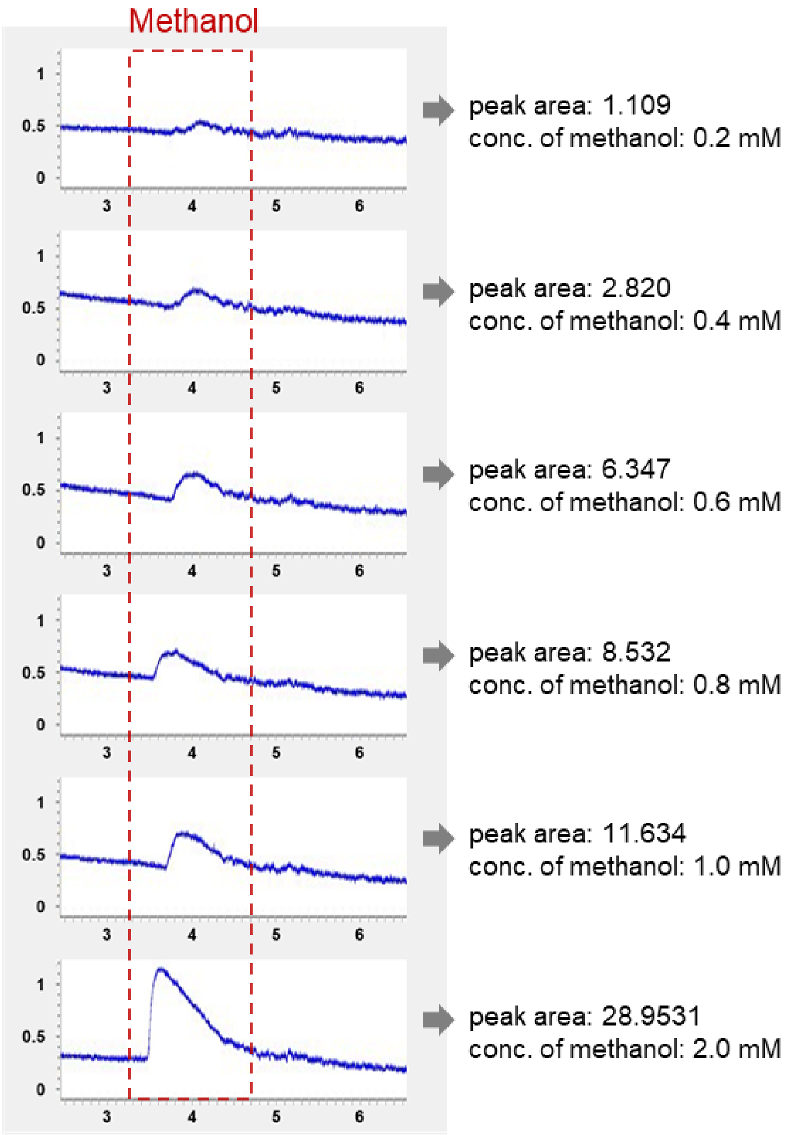

Methanol
1
0.5
0
3
4
5
6
peak area: 1.109
conc. of methanol: 0.2 mM
peak area: 2.820
conc. of methanol: 0.4 mM
peak area: 6.347
conc. of methanol: 0.6 mM
peak area: 8.532
conc. of methanol: 0.8 mM
peak area: 11.634
conc. of methanol: 1.0 mM
peak area: 28.9531
conc. of methanol: 2.0 mM

b

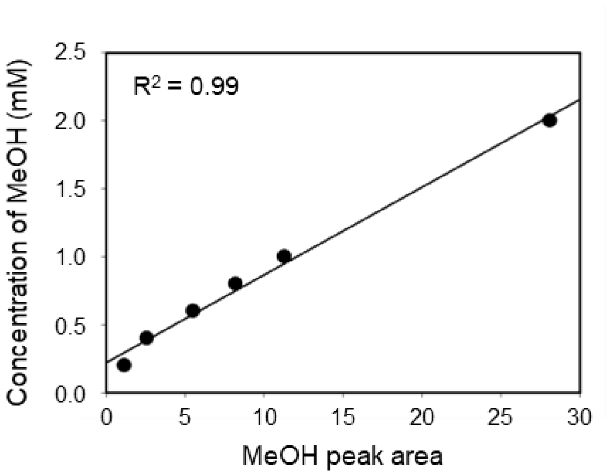

R² = 0.99
Concentration of MeOH (mM)
0.0
0.5
1.0
1.5
2.0
2.5
0
5
10
15
20
25
30
MeOH peak area

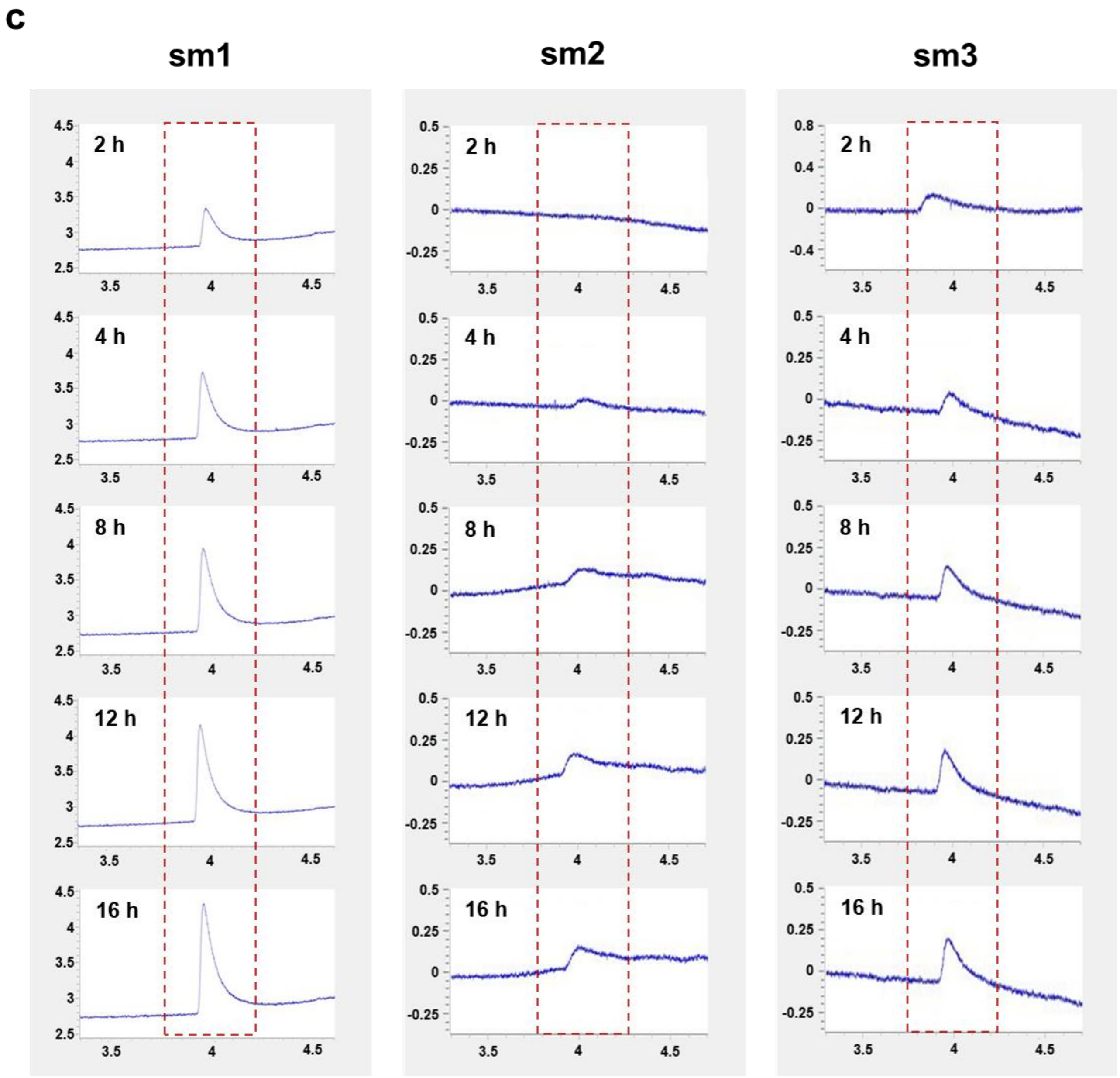

Supplementary Figure 7 | Raw data of GC analysis of methanol. (**a**) GC chromatograms for standard methanol at different concentration (0.2 to 2.0 mM). (**b**) Correlation between methanol concentration and methanol peak area of GC chromatogram of (a). (**c**) GC chromatograms of the sm1 to sm3 sampled from the methane-oxidizing solution at different time points. All of these experiments have been repeated three times. In all chromatograms of (a) and (c), the x-axis represents the analysis time (min), and the y-axis represents picoamperes (pA), with the methanol peak indicated by red dashed boxes.

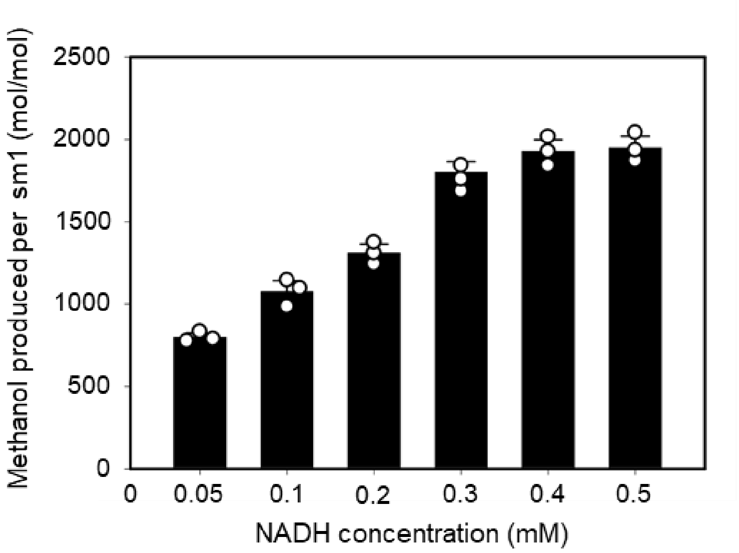

Supplementary Figure 8 | Methanol produced by sm1 at different concentrations of NADH (0.05 to 0.5 mM). Methanol concentration was measured at 16 h after the *in vitro* methane oxidation by sm1 begins. $N = 3$ independent experiments. Mean ± s.d.

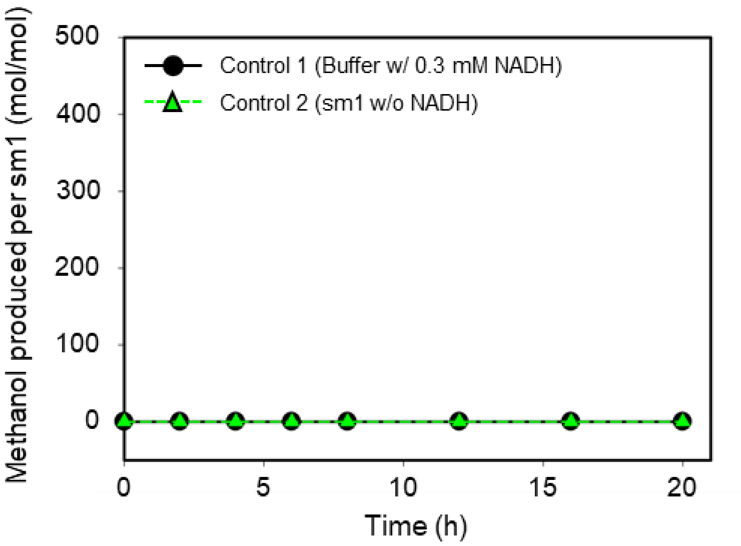

Supplementary Figure 9 | Cumulative amount of methanol produced in the two control experiments of methane oxidation: control 1 (enzyme-free buffer with 0.3 mM NADH) and control 2 (NADH-free buffer with sm1) $N = 3$ independent experiments. Mean ± s.d.

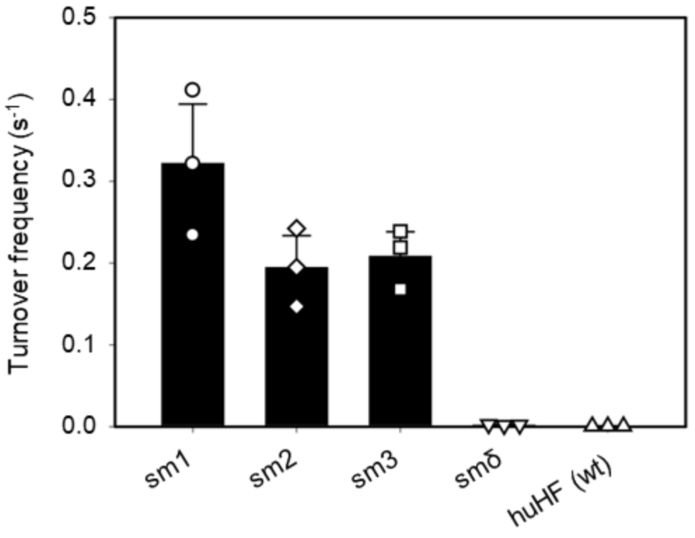

Supplementary Figure 10 | Turnover frequency of sm1 to sm3, smδ, and wild-type huHF. Turnover frequency (TF, s^{-1}) of mini-sMMOs (sm1 to sm3), smδ and wild-type huHF in the *in vitro* catalytic conversion of methane to methanol. $N = 3$ independent experiments. Mean ± s.d.

a

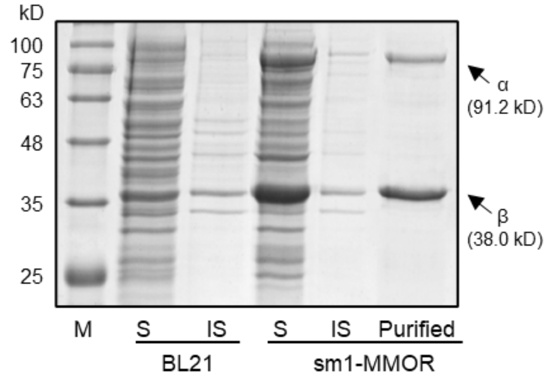

b

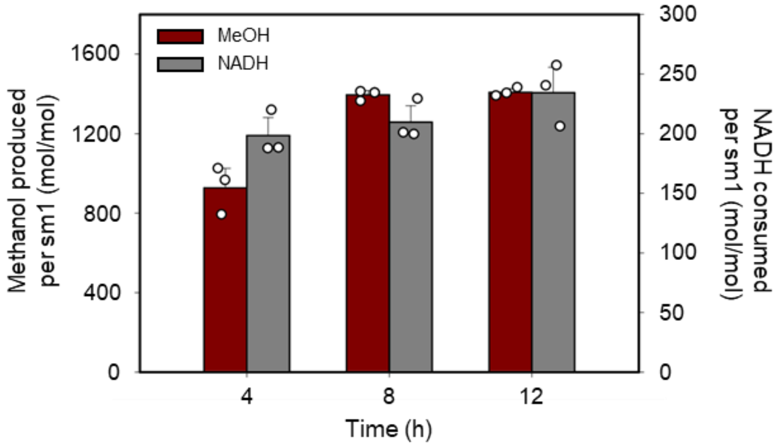

c

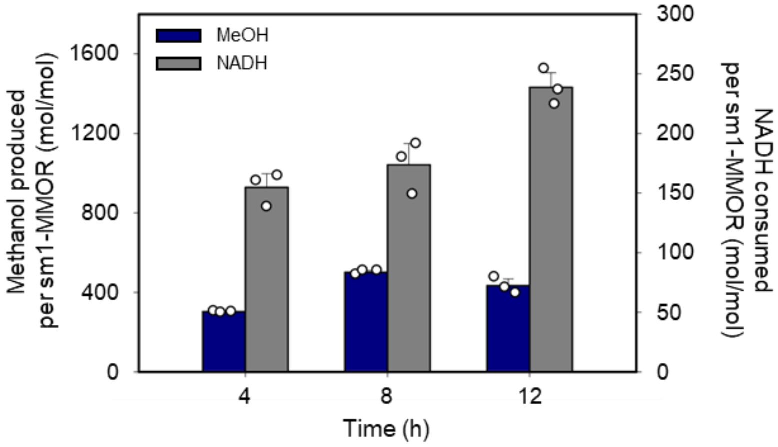

d

Coupling efficiency
(MeOH produced / NADH consumed)

Time	4 h	8 h	12 h
sm1	4.67	6.65	6.01
sm1-MMOR	1.96	2.89	1.82

Supplementary Figure 11 | Methanol-NADH coupling efficiency of sm1 and sm1-MMOR that involves a complete domain (FAD + ferredoxin domain) of MMOR. (a) SDS-PAGE analysis of soluble (S) and insoluble (IS) fraction of cell lysates from recombinant *E. coli* expressing sm1-MMOR, **(b, c)** Cumulative amount of methanol produced and NADH consumed per mole of sm1 (b) and per mole of sm1-MMOR (c), analyzed through *in vitro* methane oxidation (1.25 μM sm1/sm1-MMOR with 0.3 mM NADH) $N = 3$ independent experiments. Mean ± s.d., **(d)** Summary of coupling efficiencies calculated based on the results of (b) and (c).

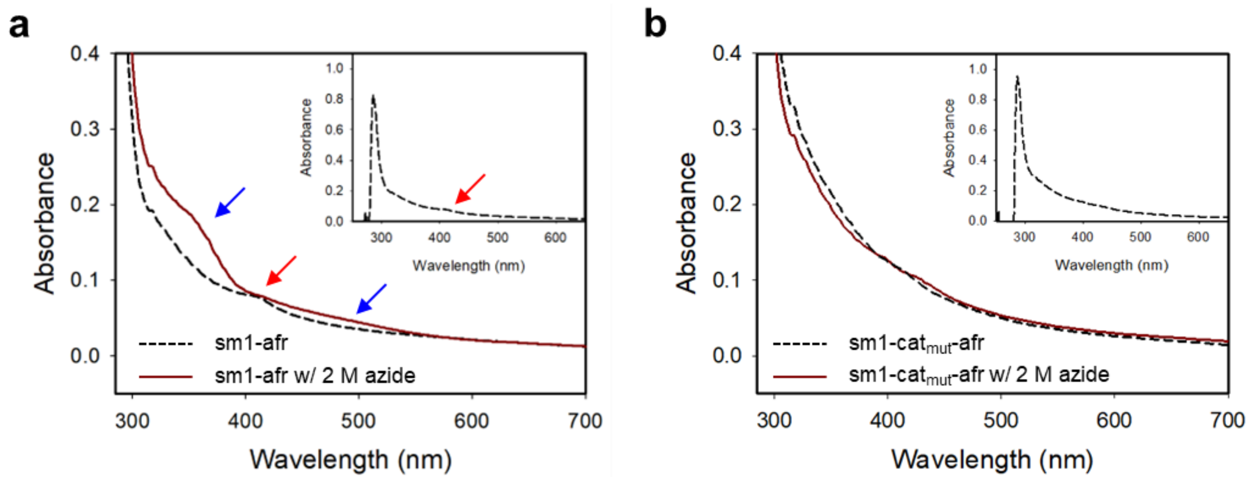

Supplementary Figure 12 | Absorption spectra from azide-diferric complex analysis with sm1-afr and sm1-cat$_{mut}$ (a) Absorption spectrum of 1.5 µM sm1-afr treated with (solid line) and without (dashed lines) 2 M sodium azide. **(b)** Absorption spectra of 1.5 µM sm1-cat$_{mut}$-afr treated with (solid line) and without (dashed lines) 2 M sodium azide. The red arrows on the dashed lines of inset and main plots indicate the peak at 395-420 nm for oxo-bridged diiron of sm1-afr[1], while the blue arrows on the solid lines of main plots indicate the peaks at 345 and 450 nm for azide-diferric ion complex[2].

a

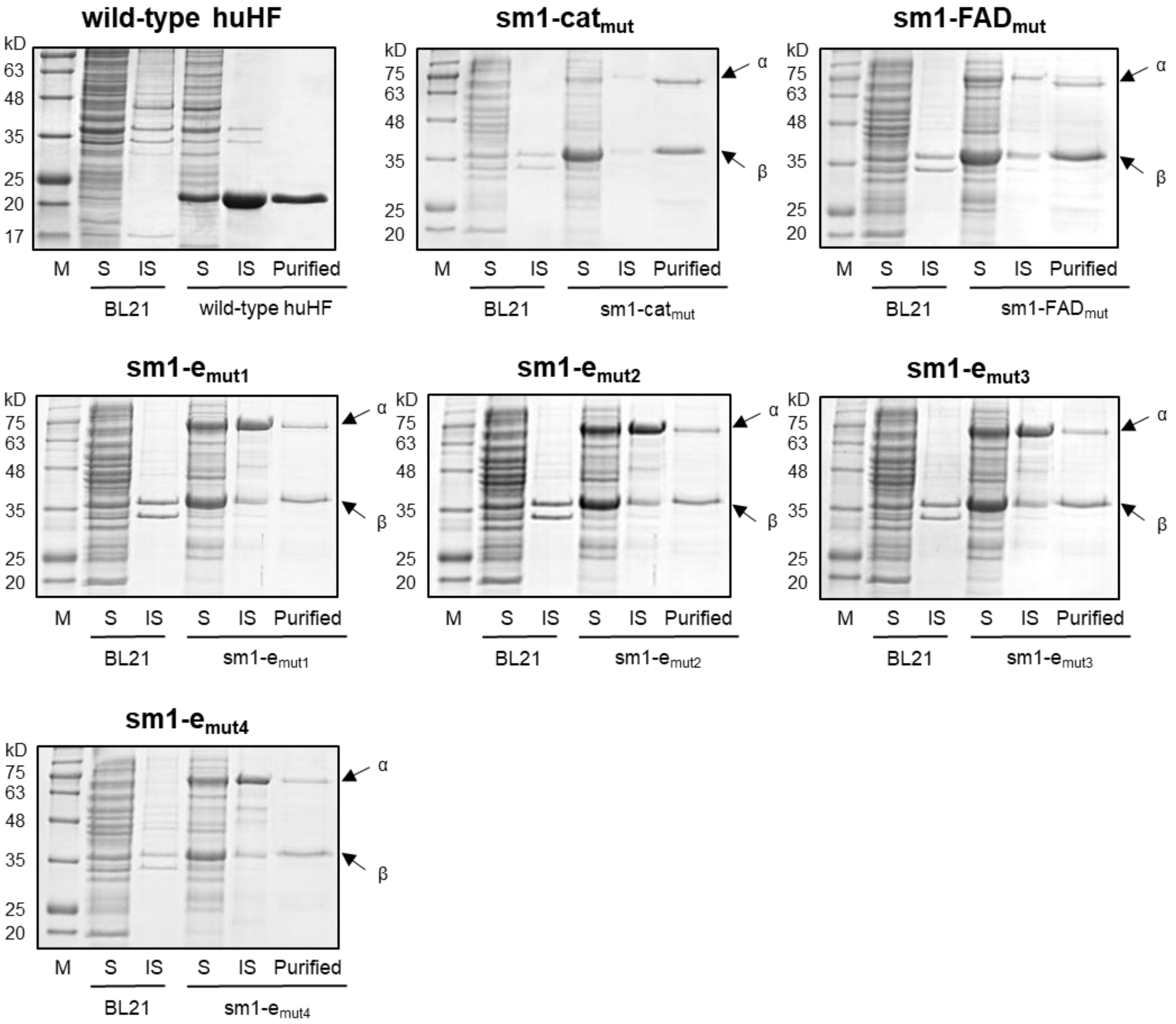
wild-type huHF
kD
63
48
35
25
20
17
M S IS S IS Purified
BL21
wild-type huHF
sm1-cat$_{mut}$
kD
75
63
48
35
25
20
α
β
M S IS S IS Purified
BL21
sm1-cat$_{mut}$
sm1-FAD$_{mut}$
kD
75
63
48
35
25
20
α
β
M S IS S IS Purified
BL21
sm1-FAD$_{mut}$
sm1-e$_{mut1}$
kD
75
63
48
35
25
20
α
β
M S IS S IS Purified
BL21
sm1-e$_{mut1}$
sm1-e$_{mut2}$
kD
75
63
48
35
25
20
α
β
M S IS S IS Purified
BL21
sm1-e$_{mut2}$
sm1-e$_{mut3}$
kD
75
63
48
35
25
20
α
β
M S IS S IS Purified
BL21
sm1-e$_{mut3}$
sm1-e$_{mut4}$
kD
75
63
48
35
25
20
α
β
M S IS S IS Purified
BL21
sm1-e$_{mut4}$

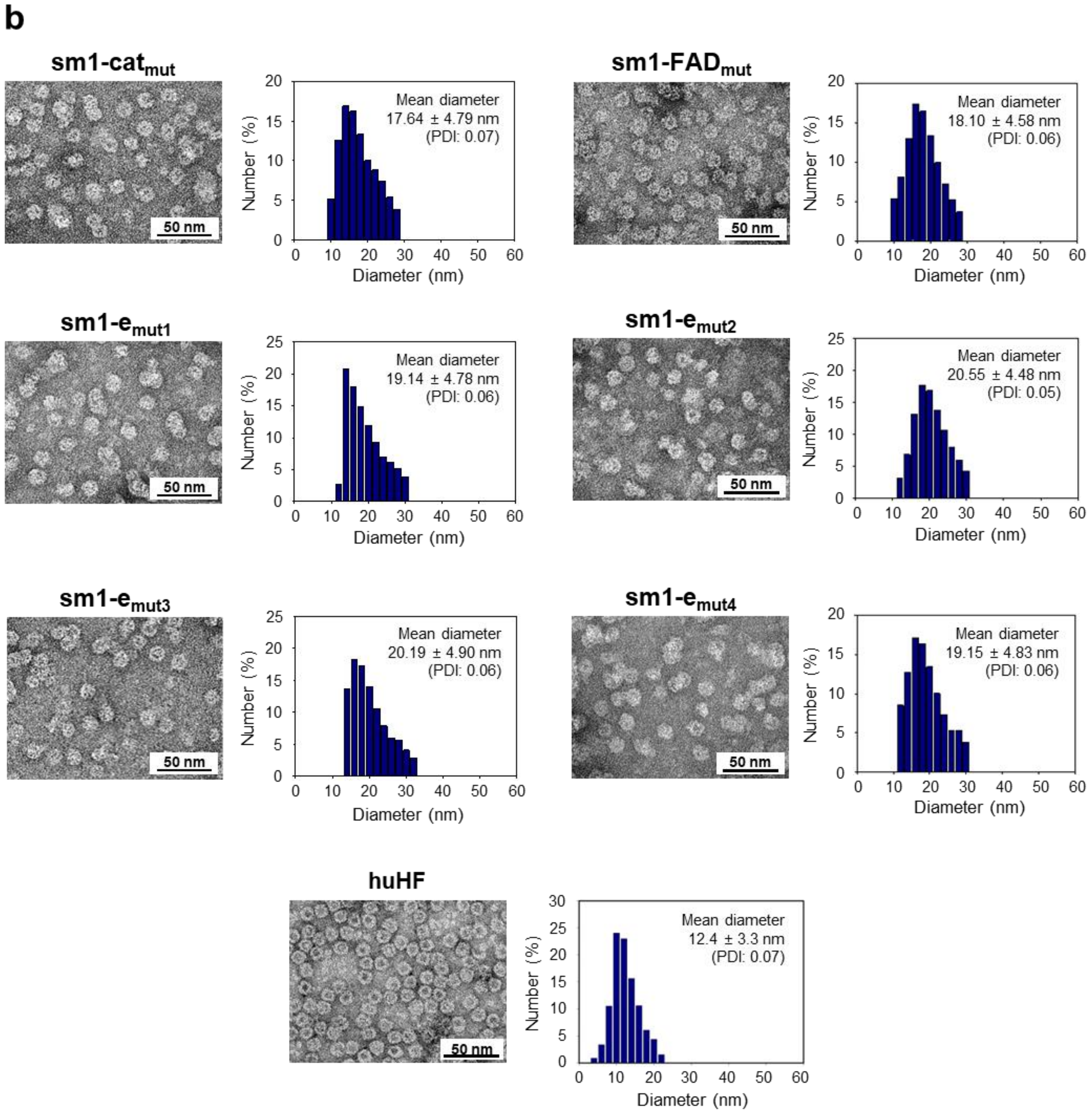

Supplementary Figure 13 | Heterologous expression of sm1-mutants and wild-type huHF. (**a**) SDS-PAGE analysis of recombinant *E.coli* expressing wild-type huHF, sm1-cat$_{mut}$, sm1-FAD$_{mut}$, and sm1-e$_{mut1}$ to sm1-e$_{mut4}$. (M: protein marker (GangNam-STAIN), S and IS: soluble and insoluble fraction of expressed protein, respectively, Purified: Ni^{2+}-affinity purification elution of soluble fraction, BL21: wild-type *E.coli* BL21(DE3)). This experiment has been repeated at least three times. (**b**) TEM images (left) and DLS data (right) of synthesized mutants (sm1-cat$_{mut}$, sm1-FAD$_{mut}$, and sm1-e$_{mut1}$ to sm1-e$_{mut4}$) and wild-type huHF. This experiment has been repeated at least three times.

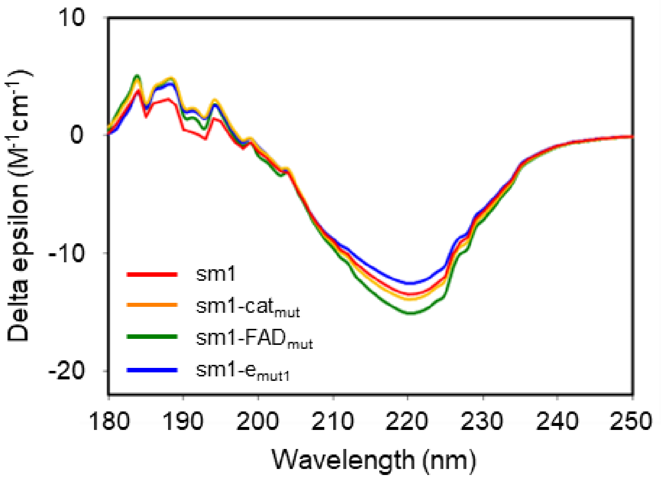

	% of each secondary structural element in sm1-mutants			
	sm1	sm1-cat_{mut}	sm1-FAD_{mut}	sm1-e_{mut1}
α-helix	43.9 (± 0.8) %	40.2 (± 6.9) %	38.9 (± 2.2) %	45.7 (± 1.5) %
β-sheet	21.1 (± 3.7) %	25.3 (± 8.9) %	28.1 (± 4.9) %	21.0 (± 2.8) %
Turn & Random coil	35.0 (± 4.4) %	34.5 (± 15.8) %	33.0 (± 7.1) %	33.3 (± 2.9) %

Supplementary Figure 14 | Results of CD spectroscopy analysis showing the secondary structures of sm1 and sm1-mutants. The table below shows the percentage (%) of secondary structural elements comprising sm1 and sm1-mutants, analyzed by CD spectroscopy. $N = 3$ independent experiments. Mean ± s.d.

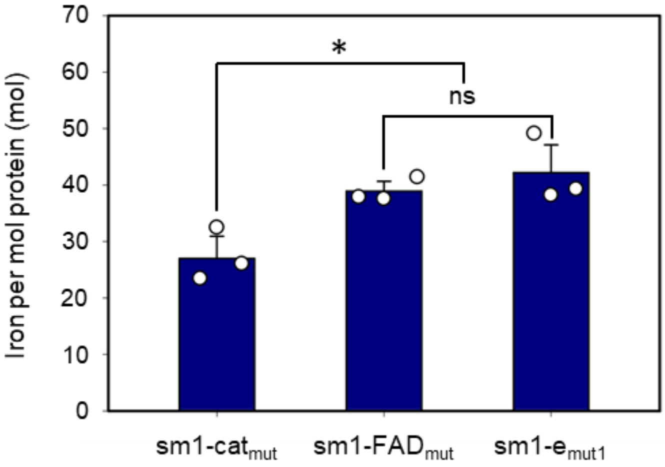

Supplementary Figure 15 | Iron contents in sm1-cat_{mut}, sm1-FAD_{mut} and sm1-e_{mut1}. $N = 3$ independent experiments. Mean ± s.d.. P-values calculated by two-tailed student's t-test (*P <0.05, ns=non-significant.).

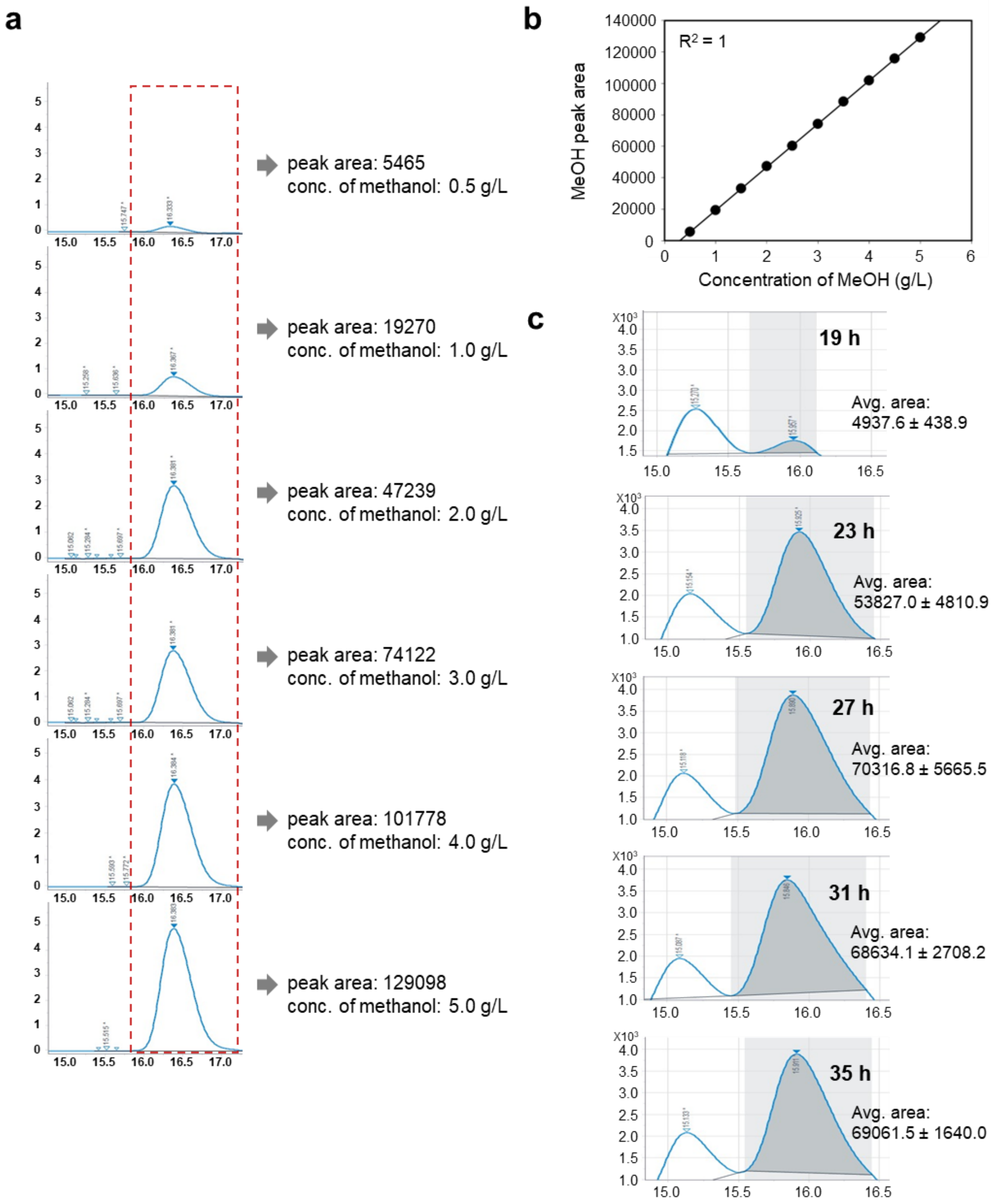

Supplementary Figure 16 | Raw data of HPLC analysis. (**a**) HPLC chromatograms of standard methanol at different concentrations (0.5 to 5.0 g/L). The red dashed box indicates the peak position of methanol. (**b**) Correlation between concentration and peak area of methanol, obtained from the HPLC chromatogram of (A). The correlation follows the equation: Y (peak area) = 27497.0 · X (methanol, g/L) - 8195.3, R^2=1. (**c**) HPLC chromatograms showing the methanol peaks (i.e. the grey colored peaks in the shaded area), obtained through the HPLC analysis of the soluble fraction of the samples taken from the methane-oxidizing fed-batch cultures of sm1-expressing *E. coli*. All of these experiments have been repeated three times. In all chromatograms of (a) and (c), the x-axis represents the analysis time (min), and the y-axis represent response (nRIU), with the methanol peak indicated by red dashed box (a) and grey colored region (c).

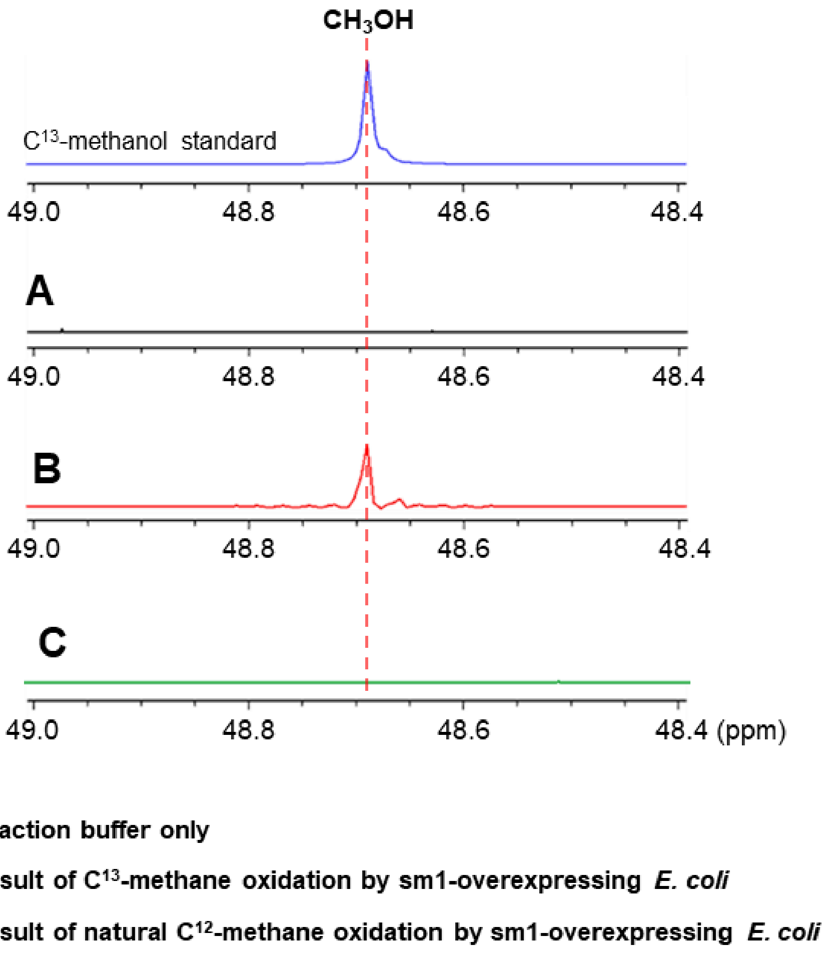

Supplementary Figure 17 | ^{13}C-NMR spectroscopy analysis to verify methane oxidation by sm1-expressing *E. coli*. For ^{13}C-NMR analysis, the whole cell-based methane oxidation was performed in a closed vial system (Methods). (A: reaction buffer only, B: Result of C^{13}-methane oxidation by sm1-overexpressing *E. coli*, and C: Result of natural C^{12}-methane oxidation by sm1-overexpressing *E. coli*). The red dashed line indicates the peak position of C^{13}-methanol.

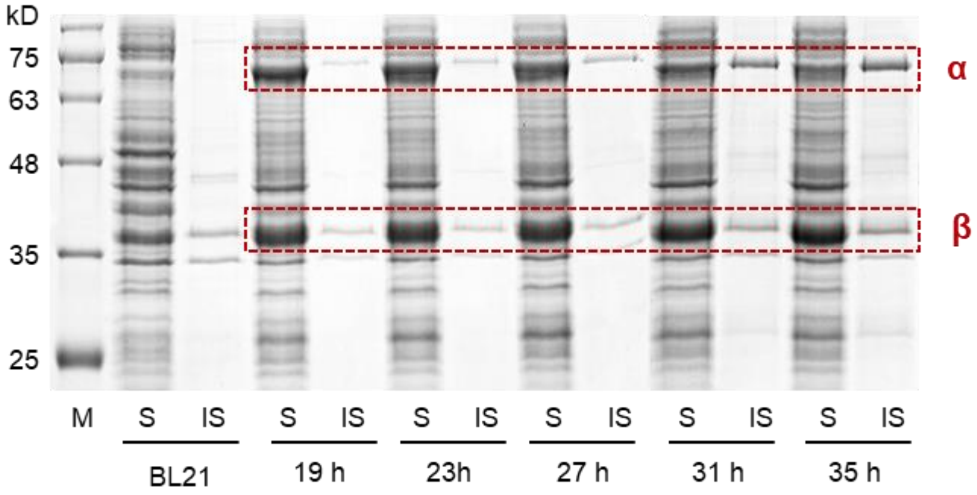

Supplementary Figure 18 | Results of SDS-PAGE of the samples taken from the fed-batch culture of sm1- expressing *E. coli*. The samples were taken at five time points after methane oxidation began by supplying methane and air mixture to the fed-batch culture in a fermenter with the working volume of 3 L. M: protein molecular marker (GangNam-STAIN), S and IS: soluble and insoluble fraction of expressed sm1, respectively, BL21: wild-type of *E. coli* BL21(DE3). The red dashed boxes indicate the size of α-subunit and β-subunit, respectively. This experiment has been repeated two times.

Supplementary References

1. Shaofeng, H. et al. Purification and Biochemical Characterization of Soluble Methane Monooxygenase Hydroxylase from *Methylosinus trichosporium* IMV 3011. *Biosci. Biotechnol. Biochem.* **71**, 122-129 (2007).
2. Makris, T. M. et al. A family of diiron monooxygenases catalyzing amino acid beta-hydroxylation in antibiotic biosynthesis. *PNAS.* **107**, 15391-15396 (2010).
