# Supplementary material for: A rationally designed miniature of soluble methane monooxygenase enables rapid and high-yield methanol production in Escherichia coli
Source: Nat Commun. 2024 May 23;15:4399. doi: 10.1038/s41467-024-48671-w (PMC11116448; doi:10.1038/s41467-024-48671-w)
Supplement: Supplementary file 3 — Description of Additional Supplementary Files [file 41467_2024_48671_MOESM3_ESM.pdf]

Description of Additional Supplementary Files

File Name: Supplementary Movie 1

Supplementary Movie 1 | MD Simulation of R_{FAD}-ΔHα. The movie shows the stable localization of FAD for 300 ns at an interface between R_{FAD} and ΔHα represented in cartoon: red wires for hydrophobic residues of R_{FAD} and ΔHα; blue wires for hydrophilic residues of ΔHα; in-between grey wires for FAD; and light violet spheres for diiron of ΔHα.
