# Supplementary material for: A rationally designed miniature of soluble methane monooxygenase enables rapid and high-yield methanol production in Escherichia coli
Source: Nat Commun. 2024 May 23;15:4399. doi: 10.1038/s41467-024-48671-w (PMC11116448; doi:10.1038/s41467-024-48671-w)
Supplement: Supplementary file 5 — Reporting Summary [file 41467_2024_48671_MOESM5_ESM.pdf]

Corresponding author(s): June Huh & Jeewon Lee

Last updated by author(s): Apr 1, 2024

Reporting Summary

Nature Portfolio wishes to improve the reproducibility of the work that we publish. This form provides structure for consistency and transparency in reporting. For further information on Nature Portfolio policies, see our Editorial Policies and the Editorial Policy Checklist.

Statistics

For all statistical analyses, confirm that the following items are present in the figure legend, table legend, main text, or Methods section.

n/a	Confirmed	
☐	☒	The exact sample size (*n*) for each experimental group/condition, given as a discrete number and unit of measurement
☐	☒	A statement on whether measurements were taken from distinct samples or whether the same sample was measured repeatedly
☐	☒	The statistical test(s) used AND whether they are one- or two-sided<br>*Only common tests should be described solely by name; describe more complex techniques in the Methods section.*
☐	☒	A description of all covariates tested
☒	☐	A description of any assumptions or corrections, such as tests of normality and adjustment for multiple comparisons
☐	☒	A full description of the statistical parameters including central tendency (e.g. means) or other basic estimates (e.g. regression coefficient) AND variation (e.g. standard deviation) or associated estimates of uncertainty (e.g. confidence intervals)
☐	☒	For null hypothesis testing, the test statistic (e.g. *F*, *t*, *r*) with confidence intervals, effect sizes, degrees of freedom and *P* value noted<br>*Give P values as exact values whenever suitable.*
☒	☐	For Bayesian analysis, information on the choice of priors and Markov chain Monte Carlo settings
☒	☐	For hierarchical and complex designs, identification of the appropriate level for tests and full reporting of outcomes
☒	☐	Estimates of effect sizes (e.g. Cohen's *d*, Pearson's *r*), indicating how they were calculated

Our web collection on statistics for biologists contains articles on many of the points above.

Software and code

Policy information about availability of computer code

Data collection	OpenLAB CDS ChemStation C.01.07 (GC), OpenLAB CDS ChemStation v2.6 (HPLC), Gatan Digital Micrograph v3.9.4 (TEM FEI-imaging software), Photal ELSZ-1000 v5.01 (DLS), NAMD2 program for performing all-atom molecular dynamics simulations, ZDOCK program for protein-protein docking simulation.
Data analysis	Quantity One v4.6.9 (SDS-PAGE), TECAN i-control v1.7 (Absorbance), Multi Gauge v2.3 (Western blot), OpenLAB CDS ChemStation C.01.07 (GC), OpenLAB CDS ChemStation v2.6 (HPLC), TopSpin 3.6.3 (NMR), Demeter v0.9.26 Athena (EXANES), Demeter v0.9.26 Artemis software (EXAFS), BeStSel program (CD spectrum, https://bestsel.elte.hu/index.php), SigmaPlot 10.0 (graph visualization), Origin 9 (XANES fitting), Pymol v2 (Protein structure visualization), Programs of gmx rms and gmx do_dssp implemented in GROMACS program for RMSD and DSSP analyses of simulated miniature sMMO model.

For manuscripts utilizing custom algorithms or software that are central to the research but not yet described in published literature, software must be made available to editors and reviewers. We strongly encourage code deposition in a community repository (e.g. GitHub). See the Nature Portfolio guidelines for submitting code & software for further information.

Data

Policy information about availability of data

All manuscripts must include a data availability statement. This statement should provide the following information, where applicable:

- Accession codes, unique identifiers, or web links for publicly available datasets
- A description of any restrictions on data availability
- For clinical datasets or third party data, please ensure that the statement adheres to our policy

The structural data for MMOR, MMOH, MMOB, and MMOH-MMOB complex are available under PDB accession numbers 1JQ4 (https://www.rcsb.org/structure/1JQ4), 1TVC (https://www.rcsb.org/structure/1TVC), 1MTY (https://www.rcsb.org/structure/1MTY), and 4GAM (https://www.rcsb.org/structure/4GAM), respectively. Sequence of MMOR, MMOH, and MMOB are available in UniProt databases under accession numbers P22868 (https://www.uniprot.org/uniprotkb/P22868), P22869 (https://www.uniprot.org/uniprotkb/P22869), and P18797 (https://www.uniprot.org/uniprotkb/P18797), respectively. The molecular dynamics simulation data of RFAD-ΔHα generated in this study is provided in Supplementary Movie1. Authors can confirm that all relevant data are included in the paper and/or its Supplementary Information files. In addition, Source Data are provided with this paper.

Research involving human participants, their data, or biological material

Policy information about studies with human participants or human data. See also policy information about sex, gender (identity/presentation), and sexual orientation and race, ethnicity and racism.

Reporting on sex and gender	N/A
Reporting on race, ethnicity, or other socially relevant groupings	N/A
Population characteristics	N/A
Recruitment	N/A
Ethics oversight	N/A

Note that full information on the approval of the study protocol must also be provided in the manuscript.

Field-specific reporting

Please select the one below that is the best fit for your research. If you are not sure, read the appropriate sections before making your selection.

☒ Life sciences ☐ Behavioural & social sciences ☐ Ecological, evolutionary & environmental sciences

For a reference copy of the document with all sections, see nature.com/documents/nr-reporting-summary-flat.pdf

Life sciences study design

All studies must disclose on these points even when the disclosure is negative.

Sample size	For experiments conducted at least three times or more, the number of independent biological repeats and technical replicates (N) are indicated in the text and figure legends with Mean ± s.d.
Data exclusions	No data were excluded from the analyses.
Replication	All experimental findings were conducted three times or more to ensure data reproducibility.
Randomization	Colonies were randomly picked for vector constructing or protein expressing. For experiments using biochemical(e.g. purified proteins) or cell samples, each sample was randomly allocated to its respective group.
Blinding	Blinding is not relevant to this study because all comparisons were performed on biochemical(e.g. purified proteins) or cell samples, with no involvement of animal or human subjects.

Reporting for specific materials, systems and methods

We require information from authors about some types of materials, experimental systems and methods used in many studies. Here, indicate whether each material, system or method listed is relevant to your study. If you are not sure if a list item applies to your research, read the appropriate section before selecting a response.

Materials & experimental systems

n/a	Involved in the study
☐	☒ Antibodies
☒	☐ Eukaryotic cell lines
☒	☐ Palaeontology and archaeology
☒	☐ Animals and other organisms
☒	☐ Clinical data
☒	☐ Dual use research of concern
☒	☐ Plants

Methods

n/a	Involved in the study
☒	☐ ChIP-seq
☒	☐ Flow cytometry
☒	☐ MRI-based neuroimaging

Antibodies

Antibodies used

monoclonal mouse anti-huHF antibody as primary antibody (ab77127, Abcam, Cambridge, UK, 1:1000 dilution), polycolonal horseradish peroxidase-conjugated goat anti-mouse secondary antibody (Cat. no. 31430, Pierce, Rockford, IL, U.S.A, 1:1000 dilution)

Validation

https://www.abcam.com/anti-ferritin-heavy-chain-antibody-ab77127.html

Plants

Seed stocks

Report on the source of all seed stocks or other plant material used. If applicable, state the seed stock centre and catalogue number. If plant specimens were collected from the field, describe the collection location, date and sampling procedures.

Novel plant genotypes

Describe the methods by which all novel plant genotypes were produced. This includes those generated by transgenic approaches, gene editing, chemical/radiation-based mutagenesis and hybridization. For transgenic lines, describe the transformation method, the number of independent lines analyzed and the generation upon which experiments were performed. For gene-edited lines, describe the editor used, the endogenous sequence targeted for editing, the targeting guide RNA sequence (if applicable) and how the editor was applied.

Authentication

Describe any authentication procedures for each seed stock used or novel genotype generated. Describe any experiments used to assess the effect of a mutation and, where applicable, how potential secondary effects (e.g. second site T-DNA insertions, mosiacism, off-target gene editing) were examined.
